# Localized delivery of therapeutics impact laryngeal mechanics, local inflammatory response, and respiratory microbiome following upper airway intubation injury in swine

**DOI:** 10.1186/s12931-024-02973-1

**Published:** 2024-09-28

**Authors:** Gabriela Gonzales, Ronit Malka, Lisa M. Marinelli, Christine M. Lee, Stacy Cook, Solaleh Miar, Gregory R. Dion, Teja Guda

**Affiliations:** 1 Department of Biomedical Engineering and Chemical Engineering, The University of Texas at San Antonio, 1 UTSA Circle, San Antonio, TX 78249 USA; 2 Department of Otolaryngology—Head and Neck Surgery, Brooke Army Medical Center, JBSA Fort Sam Houston, San Antonio, TX USA; 3 Department of Pathology and Area Laboratory Services, Brooke Army Medical Center, JBSA Fort Sam Houston, San Antonio, TX USA; 4 Department of Civil, Environmental, and Biomedical Engineering, University of Hartford, West Hartford, CT USA; 5 Department of Otolaryngology—Head and Neck Surgery,, University of Cincinnati, Cincinnati, OH USA

**Keywords:** Laryngeal injury, Endotracheal intubation, Intubation trauma, Airway microbiome

## Abstract

**Background:**

Laryngeal injury associated with traumatic or prolonged intubation may lead to voice, swallow, and airway complications. The interplay between inflammation and microbial population shifts induced by intubation may relate to clinical outcomes. The objective of this study was to investigate laryngeal mechanics, tissue inflammatory response, and local microbiome changes with laryngotracheal injury and localized delivery of therapeutics via drug-eluting endotracheal tube.

**Methods:**

A simulated traumatic intubation injury was created in Yorkshire crossbreed swine under direct laryngoscopy. Endotracheal tubes electrospun with roxadustat or valacyclovir- loaded polycaprolactone (PCL) fibers were placed in the injured airway for 3, 7, or 14 days (n = 3 per group/time and ETT type). Vocal fold stiffness was then evaluated with normal indentation and laryngeal tissue sections were histologically examined. Immunohistochemistry and inflammatory marker profiling were conducted to evaluate the inflammatory response associated with injury and ETT placement. Additionally, ETT biofilm formation was visualized using scanning electron microscopy and micro-computed tomography, while changes in the airway microbiome were profiled through 16S rRNA sequencing.

**Results:**

Laryngeal tissue with roxadustat ETT placement had increasing localized stiffness outcomes over time and histological assessment indicated minimal epithelial ulceration and fibrosis, while inflammation remained severe across all timepoints. In contrast, vocal fold tissue with valacyclovir ETT placement showed no significant changes in stiffness over time; histological analysis presented a reduction in epithelial ulceration and inflammation scores along with increased fibrosis observed at 14 days. Immunohistochemistry revealed a decline in M1 and M2 macrophage markers over time for both ETT types. Among the cytokines, IL-8 levels differed significantly between the roxadustat and valacyclovir ETT groups, while no other cytokines showed statistically significant differences. Additionally, increased biofilm formation was observed in the coated ETTs with notable alterations in microbiota distinctive to each ETT type and across time.

**Conclusion:**

The injured and intubated airway resulted in increased laryngeal stiffness. Local inflammation and the type of therapeutic administered impacted the bacterial composition within the upper respiratory microbiome, which in turn mediated local tissue healing and recovery.

**Supplementary Information:**

The online version contains supplementary material available at 10.1186/s12931-024-02973-1.

## Background

Endotracheal intubation is a common procedure for management of the upper airway but is often associated with laryngeal injury [1]. Laryngotracheal tissue injury during intubation is most often a result of mechanical trauma during endotracheal tube (ETT) insertion through the larynx, repeated irritation of the mucosal lining during prolonged intubation, or the introduction of pathogens into the respiratory tract from impaired mucociliary clearance. Complications typically present as dysphagia and dysphonia, and minor mucosal abrasions can often heal within days to weeks [2, 3]. Conversely, scar tissue formation, a natural sequelae of wound healing, can lead to functional deficits in the larynx due to the loss of vocal fold pliability [4]. Moreover, scarring in the laryngotracheal complex can result in airway narrowing, clinically referred to as stenosis, that can further impede speaking, swallowing, and breathing abilities [5, 6]. Current endoscopic and open surgical techniques for managing airway stenosis require multiple operative interventions, are typically unable to completely resolve dysphonia, and may confer high complication rates [7–9]. Therefore, therapeutic goals are shifting toward preventative measures to minimize scar tissue formation early in the treatment process. Increased insight into laryngeal mechanics, microenvironment dynamics, and the local inflammatory response to intubation injury can aid in this effort.

In addition to laryngeal injury associated with intubation, microorganisms and their self-produced extracellular polymeric matrix can attach to the surface of ETTs. Colonizing microbes within the biofilm may be pathogenic, increasing the likelihood of infections and impacting antimicrobial efficacy. The composition and matrix of the biofilm formed is variable depending on the local environment, and its microbial constituents may be impacted by inflammation. Previous investigations have demonstrated these changes in the airway microbiome and shown correlation between species and specific inflammatory insults such as asthma, cystic fibrosis, and chronic obstructive pulmonary disease [10–13]. Given that bacterial adhesion is an inevitable consequence of indwelling medical devices, and the airway is exposed to the environment and commensal bacteria, understanding the relationship between airway inflammation from intubation and microbial populations could be beneficial for establishing preventative technologies that can modulate inflammation and the microbiome. Further potentially impacting microbial populations are the impacts of medications, both topical and oral.

Various drugs are employed for airway inflammation and injuries, though current therapeutic delivery approaches are limited and, as a result, corticosteroids remain the mainstay of treatment for airway inflammation and stenosis. With the ability to locally deliver topical therapeutics over time, broader therapeutic approaches are possible. Roxadustat (FG-4592) is a prolyl hydroxylase (PHD) inhibitor that leads to the accumulation of hypoxia-inducible factors (HIF) and stimulates erythropoiesis and is approved by the FDA currently for the treatment of renal anemia [14]. Hypoxia signaling has emerged as a potential therapeutic approach for conditions such as inflammation [15, 16]. Roxadustat, which can increase local VEGF, has improved lung growth and function for potential applications for pulmonary hypoplasia in preliminary studies [17]. Preclinical studies in mice suggest it may also stabilize pulmonary fibrosis [18]. These potential therapeutic characteristics make roxadustat a useful target for deliver to the larynx and trachea.

Antiviral delivery to the upper airway has also been of increasing interest for treating respiratory infections recently [19]. One notable example of this is the use of remdesivir, an inhibitor of the viral RNA-dependent polymerase, used for treating COVID-19 [20, 21]. Another well-established antiviral with efficacy in mucosal tissues is valacyclovir, a valine derivative of acyclovir that inhibits viral deoxyribonucleic acid (DNA) polymerase [22]. Valacyclovir is widely used to treat herpes simplex virus (HSV), a common pathogen with a high infection rate that can affect mucosal membranes such as those in the respiratory tract [23]. In cases where viral infection may contribute to airway injury, such as during intubation, valacyclovir could potentially mitigate viral replication and reduce associated inflammation. Valacyclovir and related compounds have also been shown to be effective in addressing opportunistic viral pathogens in cases ranging from solid organ transplant [24] to viral positive malignancies [25]. Since the changes in airway microbiome with intubation injury and associated inflammation increase susceptibility to pathogens, there may be cause to conveniently deliver anti-viral drugs locally to site. Given that there are no definitive therapeutic approaches for treating respiratory complications, exploration of existing antiviral medications such as valacyclovir could accelerate the development of treatment regimens. Recent evidence also suggests non-oral routes useful in both drug delivery as well as treatment of respiratory infection [26, 27]. In the present study, we aimed to investigate local mechanics, inflammatory response, and microbial changes following mechanical laryngotracheal injury and the localized delivery of roxadustat and valacyclovir via drug-eluting ETT.

## Materials and methods

**Fig. 1:**
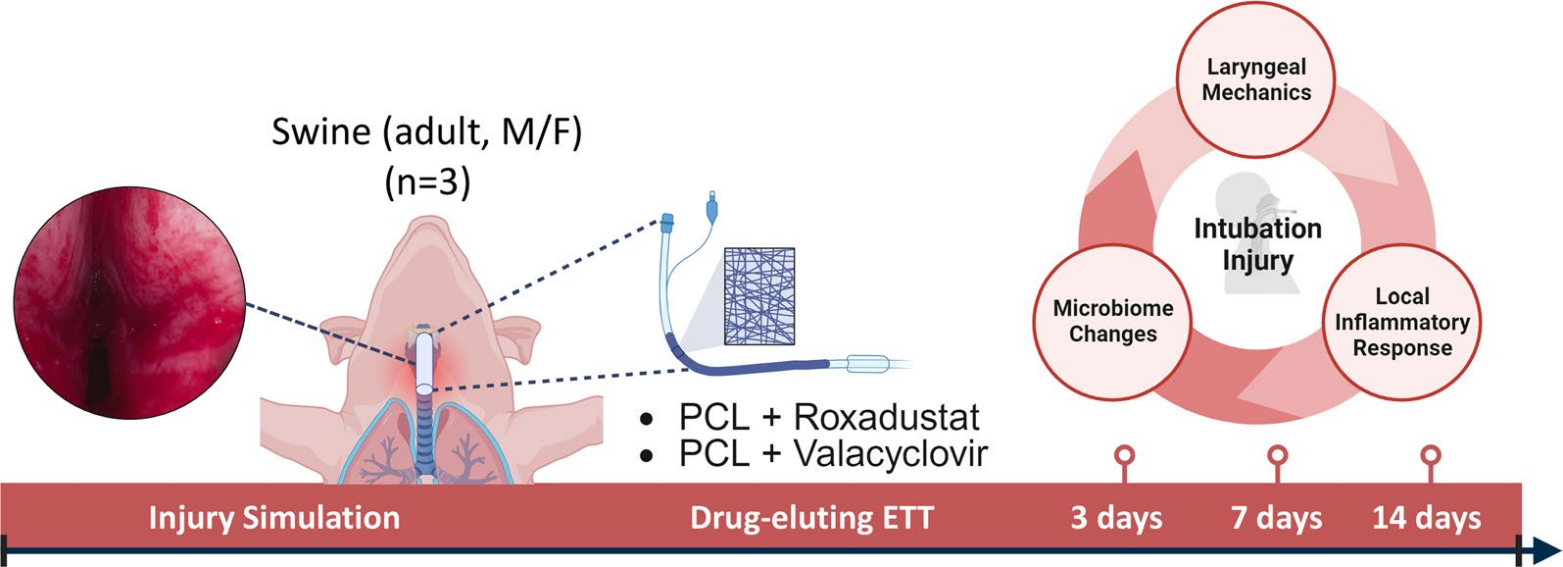
Intubation injury was simulated under endoscopic visualization and a 5 cm segment of roxadustat- or valacyclovir- loaded PCL fiber coated ETTs were placed for 3, 7, and 14 days (n = 3 per ETT type and timepoint). Laryngeal mechanics, local inflammatory response, and microbiome changes were investigated to determine how injury and local delivery of therapeutics affect outcomes

The current study was approved by the Bridge Preclinical Testing Services Institutional Animal Care and Use Committee (protocol BPTS-21-01). An experimental overview is presented in Fig. 1. Yorkshire crossbreed swine underwent direct laryngoscopy and mechanical injury to the endolarynx. Animals subsequently underwent transglottic implantation of ETT segments to simulate prolonged intubation with 9 animals receiving valacyclovir-eluting ETT segments and 9 receiving Roxadustat-eluting ETT segments. In addition, 9 animals with uncoated regular ETT placement were included to serve as a clinical standard control. Animals in each group were observed for 3 days, 7 days, or 14 days (n = 3 per timepoint), after which they were euthanized, and larynges/tracheas were excised. Localized stiffness of laryngeal tissue was evaluated with biomechanical testing. Inflammatory response associated with injury and ETT placement was investigated with immunohistochemistry and immunoassays. ETT biofilm formation was studied with scanning electron microscopy (SEM), µCT models, and histology. Finally, changes in the airway microbiome with ETT placement were assessed with 16S rRNA sequencing.

### Endotracheal tube coating

ETTs were coated via electrospinning as previously described by our group [28]. Briefly, polycaprolactone (PCL) (Mw = 80,000) was dissolved in chloroform (15:85 w/w) until homogeneous. Either valacyclovir hydrochloride (TCI Chemicals, Portland, OR) or Roxadustat (MedChemExpress, Monmouth Junction, NJ) was added to the mixture at a concentration of 10% (PCL:Drug) along with ethanol as a solvent. The solution was loaded into a Luer Lock syringe and dispensed from an 18G blunt tip needle using a syringe pump (Pump11 Elite, Harvard Apparatus, Holliston, MA) at an infusion rate of 1.8 mL/h. A 5 cm section of section of an ETT (Shiley™ Lo-Pro Oral/Nasal Tracheal Tube Cuffed, Covidien, Mansfield, MA) was positioned on a rotating rod (300 rpm) 20 cm below the needle tip where 20 kV was applied (Gamma High Voltage Research, Ormond Beach, FL). The ETTs were then sterilized with ethylene oxide prior to placement. Chemicals were purchased form Sigma-Aldrich (St. Louis, MO) unless otherwise stated.

The concentration of the drug released from the coated ETTs was evaluated over 14 days. Briefly, the absorbance of the drug in the release medium, phosphate buffered saline (PBS), was determined using a plate reader (Synergy2, Biotek, Winooski, VT) at a wavelength of 290 nm and 310 nm for valacyclovir and roxadustat, respectively. Samples (n = 5) were maintained at 37 °C and PBS was replaced fresh each day.

### Procedure and management

Animals were anesthetized via intramuscular Telazol^®^ and Ketamine 2.2 mg/kg and maintained using 0.5–5% isoflurane throughout the procedure. Analgesia was provided with Buprenorphine 0.01 to 0.05 mg/Kg. Laryngeal mechanical trauma was simulated under endoscopic visualization using a round 3/8 in. stainless steel brush. Once mucosal injury was noted in the supraglottic, glottic, and subglottic sites, a second surgeon assisted with ETT placement. A horizontal midline incision approximately 3 cm was made, and tissue was bluntly dissected until the cricothyroid membrane and the first few tracheal rings were exposed. Two 16G angiocatheter needles were passed into the airway, one through the cricothyroid membrane and the other between the first and second tracheal rings, and endoscopically visualized. A snare (Amplatz Gooseneck Microsnare Kit, Medtronic PLC, Minneapolis, MN) was then passed through each angiocatheter to the surgeon performing direct laryngoscopy to secure a 2-0 polypropylene suture through the distal end of the ETT segment. The snares were retracted through the angiocaths along with the sutures and pulled taut to advance the ETT segment into the larynx. After ensuring appropriate placement transglottically, the sutures were secured to the neck over a surgical button and the cervical incision was closed using 4-0 a polygalactin suture and Dermabond. Animals were monitored post-operatively hourly for the first four hours after surgery, every four hours for the first 24 h, and then at least twice daily until the end of the study. At the study endpoint, animals were euthanized with intravenous pentobarbital infusion (100 mg/kg) and confirmed via vital sign monitoring according to institutional protocol. The larynx and trachea were excised immediately after euthanasia, sectioned in the sagittal plane, and frozen at − 80 °C until further investigation.

### Biomechanical testing

Tissue stiffness was measured via normal indentation as previously described by our group [28–31]. Briefly, larynges were thawed at 4 °C and fixed into a Plaster of Paris sample holder. Samples were positioned beneath a camera to align a template with indentation points along the vocal fold, submerged in phosphate buffered saline (PBS), and normal indentation was conducted using a Biomomentum Mach-1 v500css (Laval, Quebec, Canada) mechanical tester with a 1.5 N uniaxial load cell. A 2 mm spherical indenter tip was used with a set velocity of 1.2 mm/s and an indentation depth of 0.3 mm. Stiffness values (N/m) were calculated from the force vs. displacement curves obtained from testing. A subset of indentation points along the vocal fold region were set aside to be evaluated across all samples.

### Histology and immunohistochemistry

Following normal indentation, laryngeal tissues were sectioned 5 mm thick along the mid-region of the vocal fold perpendicular to the mucosal surface in the supraglottis and subglottis. Samples were fixed in 4% formalin overnight and mounted in disposable embedding molds with optimal cutting temperature compound (Scigen Tissue Plus O.C.T. Compound, Thermo Fisher Scientific, Waltham, MA). A cryostat (Epredia™ NX70, Kalamazoo, MI) was used to section tissue at a thickness of 14 µm, and the sections were thaw-mounted onto glass slides, and stored at -80 °C until staining.

Slides were stained with hematoxylin and eosin (H&E) (Richard-Allan Scientific™ Signature Series™ Stains, Thermo Fisher Scientific, Waltham, MA) and Masson’s trichrome (Newcomer Supply, Middleton, WI) according to the manufacturer’s protocol. A Motic EasyScan Pro 6 Slide Scanner (Motic Instruments, Schertz, TX) was used to image slides at 20 ×. Two pathologists independently and blindly reviewed the H&E slides to assess the degree of epithelial ulceration, inflammation, and fibrosis. Both epithelial ulceration and fibrosis were graded on a five-point scale based on the percentage of epithelium ulcerated or percentage of the submucosa inferior to the vocalis muscle occupied by fibrosis, respectively (0: 0%, 1: 1–25%, 2: 25–50%, 3: 50–75%, 4: 75–100%). The degree of inflammation was graded on a three-point scale (1: mild, 2: moderate, 3: severe). Scores with differing assessments of more than one point were re-reviewed by both pathologists to achieve agreement. The area of collagen, expressed as percentage of the total area, based on Masson’s trichrome was measured in ImageJ (Version 1.53 k, National Institute of Health, USA). Briefly, images were deconvoluted using the color deconvolution2 plugin and the percentage of pixels above the threshold (0–105, min–max) in the area above the thyroid and cricoid cartilage was evaluated using the blue component representative of collagen [32]. Epithelial thickness along the vocal fold sections were also measured in ImageJ. Briefly, the straight-line tool was used to trace the scale bar, after which the known distance and units of measure were set (Analyze > Set Scale). Once the scale was calibrated, the epithelial thickness was traced using the line tool, measured (Analyze > Measure), and the values were recorded across 5 different regions per sample.

Slides were thawed at room temperature for 10 min and washed in sterile PBS to rehydrate (2 times, 10 min). They were then permeabilized in 1% goat serum and 0.4% TritonX100 in PBS (2 times, 10 min). Sections were blocked with 5% goat serum in PBS for 1 h at room temperature and placed to air dry for 5 min. Tissue sections were incubated in antibodies anti-CD86 (1:100, ab269587, Abcam, Cambridge, MA) and anti-CD206 (1:50, ab8918, Abcam, Cambridge, MA) in 5% goat serum for 2 h at room temperature. Following incubation, sections were washed with PBS (3 times, 5 min) and incubated in a secondary antibodies Alexa 647 (1:1000, Cat. A21244, ThermoFisher Scientific, Waltham, MA) and Alexa 546 (1:1000, Cat. A110033, ThermoFisher Scientific, Waltham, MA) for 1 h at room temperature. Slides were washed in PBS (3 times, 5 min), counterstained with DAPI (NucBlue™ Fixed Cell Stain, Invitrogen, Waltham, MA), washed again, and mounted with Prolong Diamond Antifade (Invitrogen, Waltham, MA). The stained slides were imaged using an Operetta CLS (PerkinElmer, Ausin, TX) with a water immersion objective lens, in non-confocal mode at 20X magnification. Images were analyzed using Harmony 4.9 PhenoLOGIC software (PerkinElmer). First, the nuclei were identified to determine the approximate number of cells. Then, the intensity properties for Alexa 647 and Alexa 546 were used to quantify the number of CD86 + and CD206 + cells and reported as a percentage of number of positive cells per total number of cells. 4 regions from the epithelium and 4 regions from the vocalis muscle were analyzed for each sample.

### Cytokine & chemokine immunoassay

The presence of IFN-α, IFN-γ, IL-1β, IL-10, IL-12/IL-23p40, IL-4, IL-6, IL-8 (CXCL8), and TNF-α were determined using a ProcartaPlex™ Porcine Panel (Invitrogen, Waltham, MA) according to the manufacturer’s protocol. Biopsy punches of the trachea were taken and 500 µL of cell lysis buffer (CellLytic™ MT, Sigma Aldrich, St. Louis, MO) was added per 100 mg of tissue along with 10 µL protease inhibitor (ThermoFisher Scientific, Waltham, MA) per 1 mL of lysis reagent. The tissue was homogenized and centrifuged at 14,000×*g* for 15 min at 4 °C. For tissue homogenates, 25 µL of Universal Assay Buffer was added to 25 µL of the sample to each well. The concentration was measured by running the samples on a Bio-Plex® 200 system using Bio-Plex manager software (Bio-Rad, Hercules, CA).

### 16S rRNA gene sequencing

At each laryngoscopy (initial and end of study), a swab of the larynx/trachea was collected and stored at -80 °C. In addition, the surface of the ETT was swabbed following their removal. The samples were given to the UTSA Genomics Core Facility to be amplified and sequenced. In short, a ZymoBIOMICS™ DNA Miniprep kit (Zymo Research) was used to extract bacterial DNA from the samples according to the manufacturer’s instructions. Following bacterial DNA isolation, the V3-V4 region of the 16S rRNA gene was amplified using universal primer sets 341F and 805R. Sequences were obtained on an Illumina MiSeq platform (Illumina, San Diego, CA) in a 2 × 300 bp paired-end run using a MiSeq v3 kit and following the 16S Metagenomic Sequencing Library Preparation protocol.

### Sequence processing

The raw sequencing reads were processed using R Studio (v2021.9.1.372, http://www.rstudio.com/). Cutadapt (v4.1) was used for removal of primers from the reads and the DADA2 pipeline (v1.16) was used for subsequent processing [33, 34]. Briefly, the demultiplexed FASTQ files for each sample were filtered and trimmed to remove low-quality sequences and run through DADA2’s core denoising algorithm to determine inferred composition of the samples. The forward and reverse reads were merged, an amplicon sequence variant (ASV) table was constructed, and chimeras were removed. Species-level taxonomy was assigned to the sequence variants using the Silva (v138.1) database [35]. The R package phyloseq, vegan, and ggplot2 were used for downstream analysis and visualization of the sequencing data [36–38].

### ETT surface characterization

SEM was used to evaluate the morphology of the ETT surfaces before and after placement. Following end of study, specimens were dried using a critical point drier (Leica, Wetzlar, Germany), sputter coated with silver-palladium (Cressington Scientific Instruments, Watford, UK), and imaged under 2 kV applied voltage at 1000 × and 5000 × magnifications using a Zeiss Crossbeam 340 Focused Ion Beam (FIB)-SEM (ZEISS, Oberkochen, Germany).

### Biofilm quantification

Following euthanasia, the extracted ETTs were placed in 10% formalin until testing. The samples were stained with phosphotungstic acid (PTA) and imaged with micro computed tomography (µCT). The ETT scans were imported into Mimics (Materialise NV, Leuven, Belguim) where the tube, biofilm, and guide wire were spatially distinguished. The volume, area, and surface area of the biofilm inclusions were determined from the segmented 3D models. Following µCT scanning, a section of the ETT was set aside for SEM.

### Statistical analysis

For comparing local stiffness, macrophage polarization, cytokine levels, alpha diversity, and biofilm formation across injured and non-injured groups, statistical analysis was performed using a two-way ANOVA (by ETT type and duration of placement) followed by Tukey’s host hoc test. For histological assessment, outcomes are reported as median (range) and differences were evaluated using Wilcoxon rank-sum test. Alpha diversity was evaluated with Shannon and Chao1 indices to estimate within-sample evenness and richness. The beta diversity describing diversity across samples was assessed with principal coordinate analysis (PCoA) based on the Bray–Curtis dissimilarity index. Statistical differences among groups were determined by permutational analysis of variance (PERMANOVA) using the function adonis from the vegan R package. Differential abundance analysis was performed with ANCOM-BC2, with p-values adjusted for multiple comparisons to control for false discovery rates [39, 40]. This adjustment was applied to comparisons between the regular ETT groups versus roxadustat and valacyclovir ETT groups and 0 days swabs versus swabs taken at 3, 7, and 14 end of study timepoints. Significant differences were determined at p < 0.05 for all statistical measures.

## Results

### ETT coating characterization

**Fig. 2:**
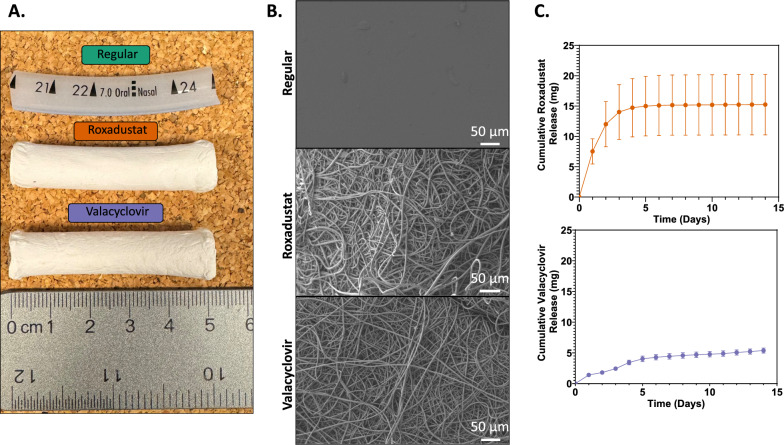
Endotracheal tube (ETT) characterization. **A** Regular, roxadustat-, and valacyclovir coated ETT segments. **B** Scanning electron microscopy (SEM) images of the drug-loaded fiber ETT coatings and **C** cumulative drug release of roxadustat and valacyclovir from ETTs over 14 days

Regular and coated ETT 5 cm segments placed in the airway are displayed in Fig. 2A. Upon observation of the ETT surface with SEM, the fiber orientation was random for both roxadustat and valacyclovir (Fig. 2B) with no observable differences between the coatings. Fiber diameter was estimated to be 4 µm for both ETT types and the average thickness of the fiber coating was 1 mm. Both drugs loaded at the same concentration produced different release profiles (Fig. 2C). Most roxadustat was released from the coating around 6 days. Valacyclovir ETTs showed sustained release over the 2-week period; however, it had a slower release rate compared to roxadustat ETTs.

### Local stiffness

**Fig. 3:**
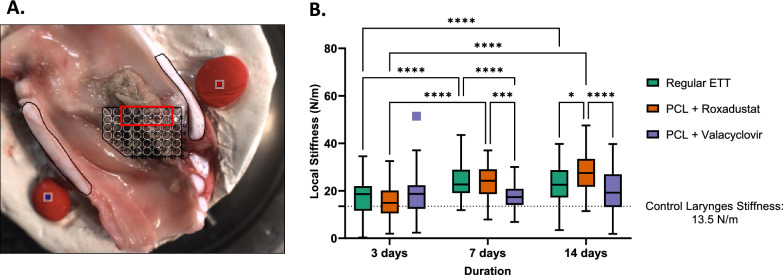
Laryngeal mechanics **A** bisected larynx with indentation points with inset region selected along the true vocal fold for analysis. **B** Local stiffness outcomes of laryngeal tissue following injury and placement of regular, roxadustat-, or valacyclovir- coated ETTs for 3, 7 and 14 days. Control larynges without ETT placement or injury specified by dashed line. Statistically significant differences are indicated by * < 0.05, ** < 0.01, *** < 0.001, and **** < 0.0001

Localized stiffness outcomes are summarized in Fig. 3. In animals with regular ETT placement, vocal fold stiffness after mechanical injury significantly increased with longer intubation time from 17.0 ± 0.757 N/m at 3 days, to 24.1 ± 0.680 N/m at 7 days (p < 0.001), and 23.1 ± 0.725 N/m at 14 days (p < 0.001). A similar trend was observed in tissue with roxadustat ETT placement with significant stiffness increase from 15.0 ± 0.992 N/m at 3 days, to 23.7 ± 0.906 N/m at 7 days (p < 0.001), and 27.3 ± 1.03 N/m at 14 days (p < 0.001). Tissue treated with valacyclovir ETT placement did not have stiffness changes over time, going from 18.6 ± 1.09 N/m at 3 days, to 17.6 ± 0.636 N/m at 7 days, and 19.7 ± 1.11 N/m at 14 days. When comparing the type of ETT used, roxadustat ETT groups had significantly greater vocal fold stiffness than regular ETTs at 14 days (p = 0.01) and valacyclovir ETT groups at 7 (p < 0.001) and 14 days (p < 0.0001). Valacyclovir ETTs presented significantly lower stiffness outcomes than regular ETTs at 7 days (p < 0.001), but there was otherwise no significant difference in stiffness at any other timepoint.

### Histologic analysis

**Fig. 4:**
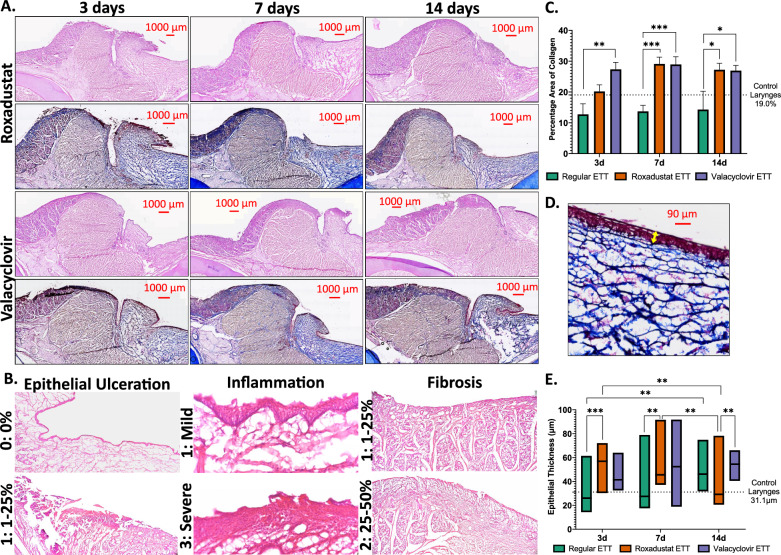
H&E and Masson’s Trichrome stained cross-sections **A** Vocal fold tissue with roxadustat- or valacyclovir- coated ETT placement after 3, 7, or 14 days. **B** Magnified regions of vocal fold demonstrating different grades of epithelial ulceration (40 ×), inflammation (400 ×), and fibrosis (40 ×) determined by pathologists. **C** Area of collagen, expressed as percentage of the total area. **D** Magnified region of vocal fold illustrating epithelial thickness measurement depicted by yellow arrow. **E** Quantification of epithelial thickness. Dashed line represents measurements for control larynges without ETT placement or injury. Statistically significant differences are indicated by * < 0.05, ** < 0.01, *** < 0.001, and **** < 0.0001

Epithelial ulceration showed a median score of 1 at all timepoints with roxadustat-coated ETTs, compared to a median of 1 at 3 days and 0 at 7 and 14 days for valacyclovir-coated ETTs (Table 1). The extent of fibrosis was similarly unchanged between timepoints, with median scores of 1 at all timepoints for the roxadustat group and 1–2 for the valacyclovir group. The degree of inflammation demonstrated a trend toward persistence in the roxadustat treated group, with a median score of 3 (rated as severe) at all timepoints. By contrast, the valacyclovir-coated ETTs showed a trend toward reduced inflammation over time, with a score of 3 at day 3 and a score of 1 at subsequent time points. However, with nonparametric testing none of these comparisons reached statistical significance. Histology outcomes are presented in Fig. 4.

**Table 1:**
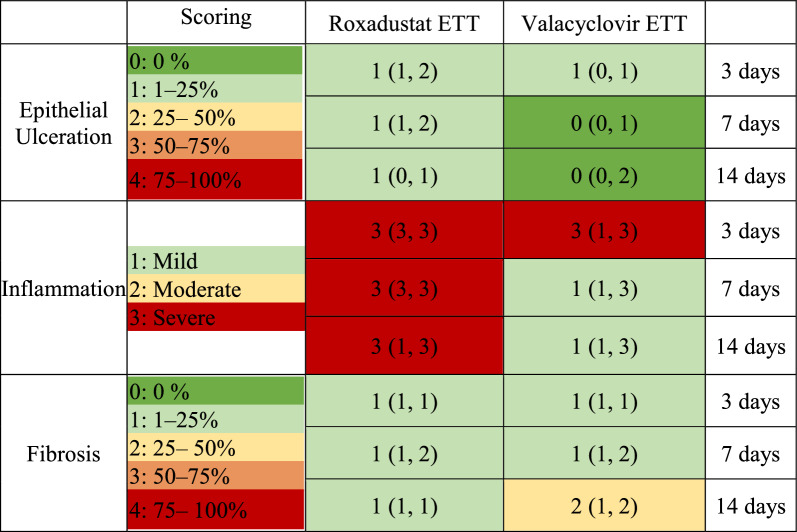
Summary of histology scoring metrics (left) and median and range scores injured airways with endotracheal tube placement (right)

Note: Table color coding represents the severity of epithelial ulceration, inflammation, and fibrosis on a heat map ranging from green (less severe) to red (more severe). Epithelial ulceration and fibrosis were graded on a five-point scale: 0: 0%, 1: 1–25%, 2: 25–50%, 3: 50–75%, 4: 75–100%. Inflammation was graded on a three-point scale: 1: mild, 2: moderate, 3: severe.

There was a significant difference in the percentage of collagen between the type of ETT placed in the injured airway (p < 0.001). Laryngeal tissue with roxadustat coated ETT placement had a significantly greater percentage of collagen at 7 (30.1 ± 2.13%, p < 0.01) and 14 days (27.2 ± 2.13%, p = 0.010) than with regular ETTs (13.7 ± 2.13% and 14.3 ± 3.37%, respectively). Similarly, groups with valacyclovir coated ETTs had a significantly greater percentage of collagen in comparison to regular ETT groups at 3 (27.3 ± 2.38%, p = 0.005), 7 (28.9 ± 2.75%, p < 0.001), and 14 days (26.9 ± 2.75%, p = 0.022). Investigation of epithelial thickness determined significant differences between ETT types over time (p < 0.001). The epithelial thickness of vocal fold tissue with regular ETT placement significantly increased from 3 (33.5 ± 3.51 µm) to 14 days (49.2 ± 3.69 µm, p = 0.006). Tissues with roxadustat ETT placement had a significant decrease in epithelial thickness from 3 (53.5 ± 3.69 µm) to 14 (37.9 ± 3.69 µm, p = 0.008) and 7 (54.0 ± 3.30 µm) to 14 days (p = 0.003). The epithelial thickness was significantly greater in groups with roxadustat ETT versus regular ETT placement at 3 (p < 0.001) and 7 days (p = 0.003). At 14 days, the epithelial thickness was significantly greater in tissue treated with valacyclovir ETTs in comparison to roxadustat ETTs (p = 0.004).

### Local inflammatory response

**Fig. 5:**
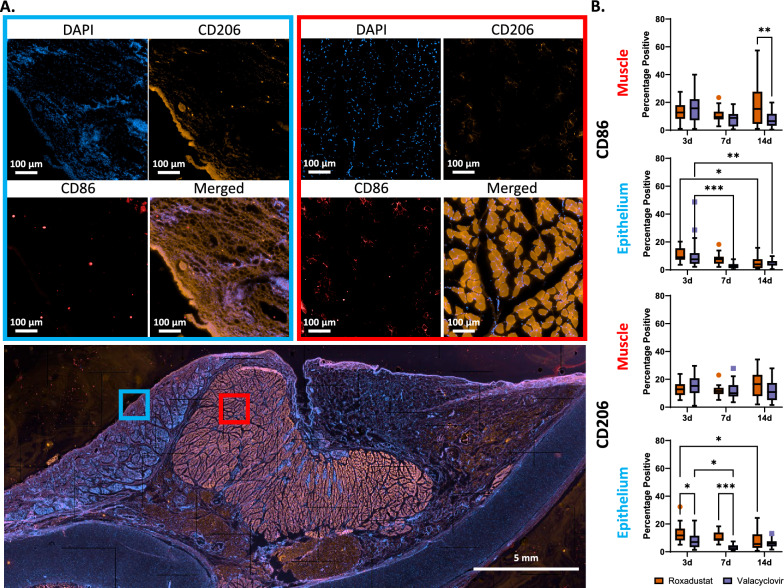
Immunostaining of CD86 (red fluorescence) and CD206 (orange fluorescence) **A** Representative stained vocal fold section with inset of epithelial and vocalis muscle regions **B** Quantification of surface marker expression determined from immunohistochemistry. Statistically significant differences are indicated by * < 0.05, ** < 0.01, *** < 0.001, and **** < 0.0001

**Fig. 6:**
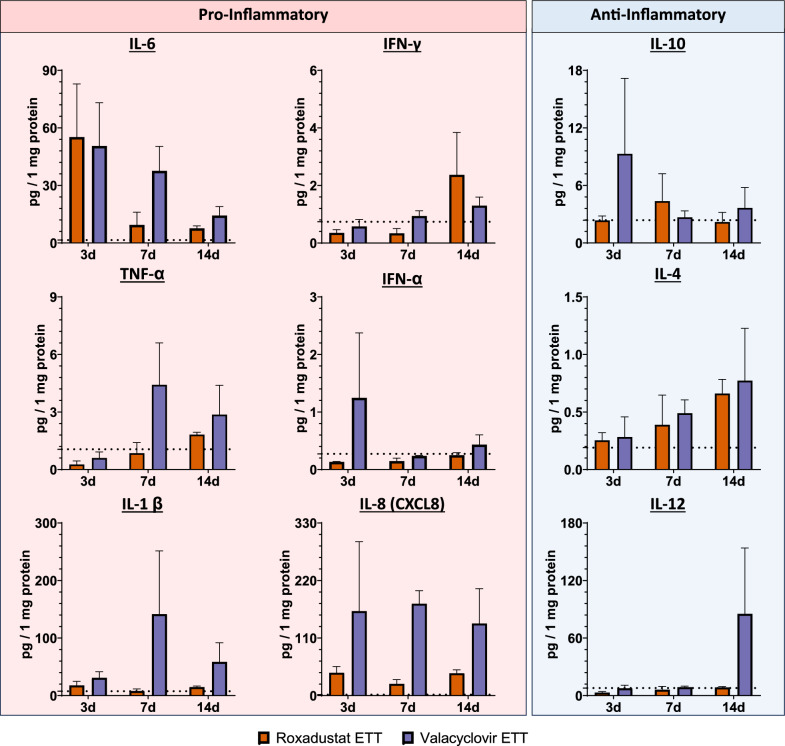
Laryngeal tissue levels of IL-6, IFN-γ, TNF-α, IFN-α, IL-1 β, IL-8 (CXCL8), IL-10, IL-4, and IL-12. Baseline levels of the markers in uninjured and untreated animals are indicated by the dotted lines: IL-6 (1.53 pg/mg), IFN-γ (0.74 pg/mg), TNF-α (1.06 pg/mg), IFN-α (0.27 pg/mg), IL-1β (7.70 pg/mg), IL-8 (2.05 pg/mg), IL-10 (2.37 pg/mg), IL-4 (0.19 pg/mg), IL-12 (7.86 pg/mg)

We examined the presence of CD86 and CD206 positive cells, M1 and M2 macrophage respectively (Fig. 5). In the vocalis muscle, roxadustat treated tissue presented significantly higher M1 macrophage in comparison to valacyclovir treated tissue at 14 days (p = 0.002). However, there were negligible differences in anti-inflammatory M2 macrophage in the same region between treatment types and time. There was a significant decrease of M1 macrophage in epithelial tissue with roxadustat ETT placement from 3 to 14 days (p = 0.024). Groups with valacyclovir ETT placement had significant decreases in M1 macrophages from 3 to 7 days (p = 0.0004) and 3 to 14 days (p = 0.004). This trend remained the same for M2 macrophage observations with roxadustat treated epithelium significantly decreasing from 3 to 14 days (p = 0.012) and valacyclovir treated epithelium significantly decreasing from 3 to 7 days (p = 0.043). Laryngeal tissue with roxadustat ETT placement reported significantly higher anti-inflammatory marker percentage than with valacyclovir ETT placement at 3 and 7 days (p = 0.039 and p < 0.001 respectively). Evaluation of inflammatory cytokines and chemokines is shown in Fig. 6**.** No statistically significant differences were observed across ETT types or duration of placement, aside from IL-8. IL-8 levels differed significantly between the roxadustat and valacyclovir ETT groups (p = 0.009), with IL-8 levels remaining consistent over time in both groups but higher in the valacyclovir-treated animals. While no other cytokines demonstrated statistically significant differences across treatment type or time, certain trends were observed. IL-6 and IL-4 exhibited an inverse trend over time, with IL-6 levels trending downward and IL-4 levels trending upward in both ETT groups. In the valacyclovir group, there was a trend of elevated levels of IL-10 and IFN-α observed at 3 days before returning closer to native control levels at later time points. TNF-α and IL-1β levels peaked at 7 days in the valacyclovir group, whereas the roxadustat group remained close to control levels throughout the study. At 14 days, IFN-γ levels appeared elevated in the roxadustat group and IL-12 levels were also elevated in the valacyclovir group, although without statistical significance.

### Laryngeal microbiome changes before and after injury

A swab of the larynx was taken at initial laryngoscopy before injury (0d), at the end of study, and of the surface of the ETT following their removal. Sequencing of the V3-V4 region of the 16S rRNA gene produced an average of 65,996 reads per sample. After processing sequences with the DADA2 pipeline, there was a total of 2,406,257 remaining reads with 6,757 unique sequence variants identified. Overall, there was a total of 23 phyla, 39 classes, 86 orders, 152 families, and 396 genera recognized.

**Fig. 7:**
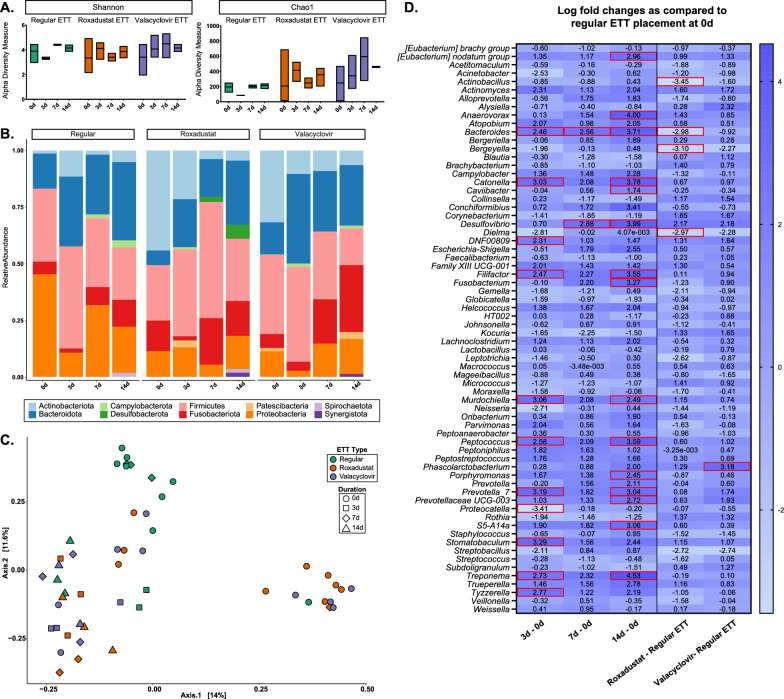
Microbial changes of larynx with injury and localized delivery of roxadustat and valacyclovir via ETTs **A** Alpha diversity measure based on Shannon and Chao1 indices **B** mean relative abundance at the phylum level **C** beta diversity analysis visualized with principal coordinate analysis (PCoA) based on Bray–Curtis dissimilarity index and **D** differential abundance analysis assessed with ANCOMBC2. Significant differentially abundant bacteria are highlighted red

Microbial composition for laryngeal tissue swabs before injury and after end of study are summarized in Fig. 7. There was no significant difference in alpha diversity as measured by the Shannon index across ETT type and duration of placement, however, Chao1 indices indicated significance between ETT type (p = 0.01). The top 5 phyla representing the most abundance across airway swabs were Firmicutes (31.9%), Bacteroidota (19.5%), Proteobacteria (17.9%), Actinobacteriota (17.4%), and Fusobacteriota (10.3%). After 3 days the predominant genera were *Peptostreptococcus* and *Porphyromonas* for all ETT Types. At 7 days predominant genera were *Actinobacillus* and *Alloprevotella* for the groups with regular ETT placement, *Peptostreptococcus* and *Fusobacterium* for the groups with roxadustat ETT placement, and *Fusobacterium* and *Porphyromonas* for groups with valacyclovir ETT placement. After 14 days, the primary genera were *Porphyromonas* and *Fusobacterium* for all ETT Types. The PCoA based on Bray–Curtis dissimilarity showed 25.6% of total variance between samples. The first and second principal coordinates distinguished similarity between Roxadustat, valacyclovir, and late timepoints for the Regular ETT groups. Consistent with these observations, the PERMANOVA test indicated statistically significant differences in the microbial composition with ETT Type (R^2^ = 0.10, p < 0.05) and duration of placement (R^2^ = 0.13, p < 0.05). Differential abundance analysis identified significant changes in several genera including negative log fold changes in *Actinobacillus* (adjusted p = 0.022)*, Bacteroides* (adjusted p < 0.0001)*, Bergeyella* (adjusted p = 0.001), and *Dielma* (adjusted p = 0.027) for groups with roxadustat coated ETT groups in comparison to regular ETT and significant positive log fold changes in *Phascolarctobacterium* (adjusted p < 0.0001) in groups with valacyclovir coated ETT placement. Additionally, 29 other significant taxa were identified, with detailed p-values provided in Supplementary Table 1.

**Fig. 8:**
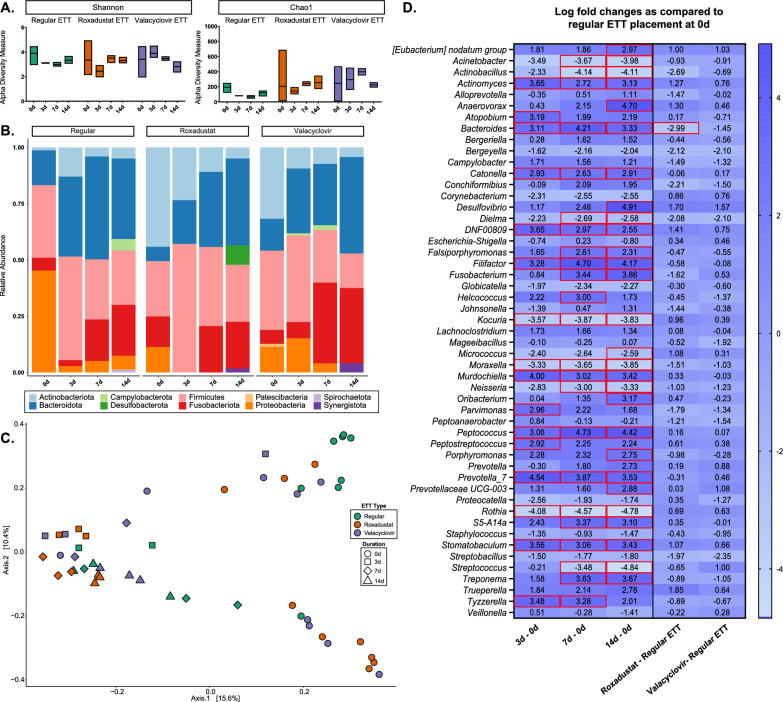
Microbial changes observed on the surface of coated ETTs **A** Alpha diversity measure based on Shannon and Chao1 indices **B** mean relative abundance at the phylum level **C** beta diversity analysis visualized with principal coordinate analysis (PCoA) based on Bray–Curtis dissimilarity index and **D** differential abundance analysis assessed with ANCOMBC2. Significant differentially abundant bacteria are highlighted red

When considering microbial composition before injury and on the surface of the ETTs (Fig. 8), there were similar outcomes for within-sample variance with higher Shannon and Chao1 indices recorded in coated ETTs than uncoated ETTs. Analysis of Shannon index yielded no significant differences in alpha diversity while Chao1 richness showed significant differences between ETT type (p < 0.03). The top 5 most abundant phyla on the ETT surface were Firmicutes (31.0%), Bacteroidota (22.7%), Actinobacteriota (17.9%), Fusobacteriota (13.0%), and Proteobacteria (12.8%). *Peptostreptococcus* was amongst the predominant genera identified on the surface of ETTs after 3 days along with *Prevotella_7* for regular ETTs, *Actinomyces* for roxadustat ETTs, and *Porphyromonas* for valacyclovir ETTs. After 7 days the main genus seen on the surface of the ETTs was *Fusobacterium*, while regular ETTs also contained *Porphyromonas* and roxadustat and valacyclovir ETTs had *Peptostreptococcus*. As identified in the airway genera, *Fusobacterium* and *Porphyromonas* were the top genera on the surface of all ETT types after 14 days. The PCoA showed 26.0% total variance between samples, with ETT groups across all timepoints clustering together and exhibiting distinction from the microbiome before injury and ETT placement. This was supported by PERMANOVA analysis which determined differences in community composition with ETT type (R^2^ = 0.08, p < 0.05) and duration (R^2^ = 0.17, p < 0.05). When considering ETT type, the roxadustat coated ETT yielded negative log fold changes of *Bacteroides* (p = 0.0006) when compared to the regular ETT surface. Notably, the ETT surface contained significantly positive log fold changes of *Anaerovorax* (adjusted p < 0.0001), *Desulfovibrio* (adjusted p < 0.0001)*, Filifactor* (adjusted p < 0.0001)*, Fusobacterium* (adjusted p = 0.0002)*, Peptococcus* (adjusted p < 0.0001)*, Prevotella_7* (adjusted p = 0.006), and *Treponema* (adjusted p = 0.0002) at 14 days when compared to swabs taken before injury and placement. Additionally, 60 other significant taxa were identified, with detailed p-values provided in Supplementary Table 2.

### ETT biofilm

**Fig. 9:**
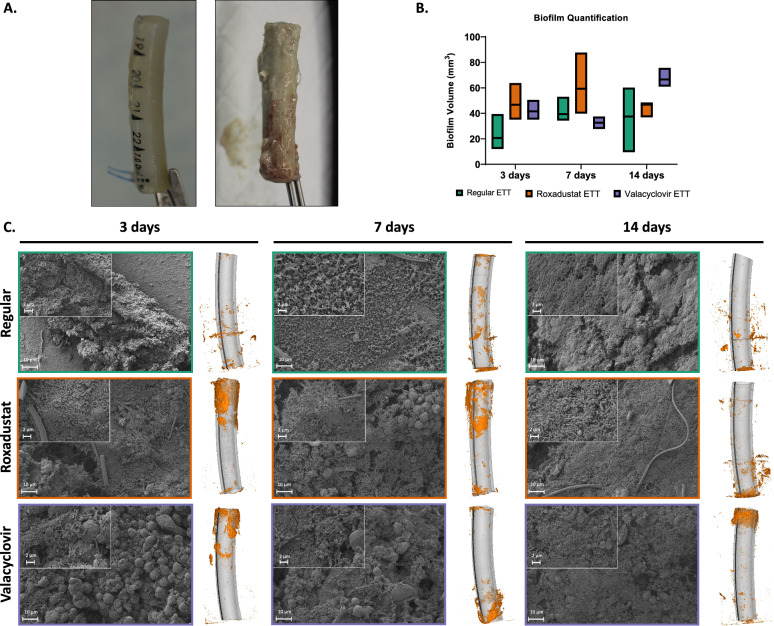
Endotracheal tubes after end of study **A** Regular ETT and coated ETT at 14-day timepoint demonstrating biofilm formation, **B** biofilm quantification, and **C** SEM images and µCT composites of regular, roxadustat-, and valacyclovir- coated ETTs following 3, 7, and 14 days of placement

SEM demonstrated an abundance of bacteria on the coated ETT surface (Fig. 9). Analysis of the composites based on µCT determined higher biofilm inclusions for the coated ETTs than the regular ETTs, however, there was no statistical significance. Regular ETTs had an increase in biofilm formation from 3 (24.0 ± 6.59 mm^3^) to 7 (42.2 ± 4.54 mm^3^) days and a decrease from 7 to 14 (35.8 ± 12.0 mm^3^) days. Roxadustat ETTs demonstrated the same trend with an increase from 3 (48.5 ± 6.78 mm^3^) to 7 (62.3 ± 11.3 mm^3^) days and decrease from 7 to 14 (44.1 ± 2.97 mm^3^) days. Valacyclovir ETTs had a decrease in biofilm from 3 (42.4 ± 3.65 mm^3^) to 7 (32.6 ± 3.41 mm^3^) days and increased to the greatest volume at 14 days (67.8 ± 3.49 mm^3^).

## Discussion

The vocal folds are central to generating sound through vibration and airflow modulation. Damage to the mucosal interface or superficial lamina propria under the delicate epithelium significantly impairs vocal fold function and phonation, emphasizing the need to better understand how pathological insults such as traumatic intubation and subsequent therapeutic interventions impact mechanics to optimize treatment approaches. Studies using shear rheometry to evaluate the viscoelastic properties of the vocal fold after injury found that scarred tissue exhibited higher elastic shear modulus and dynamic viscosity compared to non-scarred tissue [41, 42]. Our group has previously investigated localized stiffness of vocal fold tissue after recurrent laryngeal nerve injury utilizing normal indentation and found biomechanical properties to correlate with vocalis muscle atrophy [31]. In this study examining the delivery of roxadustat and valacyclovir in the injured airway, we found local laryngeal tissue stiffness increased as the length of intubation progressed for regular and roxadustat ETTs but remained consistent across all time-points for valacyclovir ETTs. Although valacyclovir is primarily known for its antiviral effects, it produced stiffness outcomes closest to the healthy control at 14 days. Moreover, valacyclovir ETTs demonstrated a reduction in epithelial ulceration and inflammation and a statistically insignificant increase in fibrosis over time on histopathological assessment. Other studies utilizing corticosteroids for the modulation of inflammation in inhalational burn and intubation injury demonstrated similar outcomes indicating valacyclovir may also modulate the immune response [28, 43].

Amongst the extracellular matrix that contribute to the vocal fold’s vibratory function are collagen and elastin [44–46]. After injury, the extent of collagen deposition as part of wound healing stabilizes at 3 weeks. Although collagen remodeling continues for months, normal vocal fold viscoelasticity is never fully restored [44, 47]. Characterization of vocal fold scarring in other animal models established that scar tissue and subsequent stiffness outcomes were likely associated with the disorganized nature of the collagen fibers during tissue remodeling rather than from an increased amount of collagen deposition [41, 42, 48]. Local delivery of therapeutics may vary collagen deposition outcomes as demonstrated in another study utilizing drug-coated stents for the treatment of tracheal granulation in rabbits that showed localized delivery of antibiotics reduced collagen density [49]. Our group’s previous investigation of collagen deposition after intubation injury and the delivery of dexamethasone via a coated ETT showed an extent of collagen deposition similar to that observed in regular ETTs, which was less than that of control tissue [43]. In the present study’s assessment of the extent of collagen deposition, roxadustat and valacyclovir ETTs yielded higher measurements than regular ETTs and control larynges. At 3 days, tissue with roxadustat ETT placement had the closest resemblance to uninjured control vocal fold tissue. Because the distinction between collagen fiber types was not possible with our histological methods, further investigations will focus on evaluating the nature of the collagen present in order to better characterize differences in the remodeling process with different therapeutic options.

The vocal fold’s epithelium serves as a protective barrier against mechanical insults and disruption puts the tissue at risk of damage and bacterial infection. Complete re-epithelialization has been shown as early as 5 days after injury in a rabbit model of acute vocal fold wound healing [50]. Another epithelial injury model in rats found that despite restoration after 3 days post-injury, the functional integrity was not fully restored [51]. To further complicate mucosal remodeling, continuous contact between an ETT and the laryngotracheal epithelium during intubation may further impede healing after injury. We observed an increase in thickness of the vocal fold epithelium over time for regular and roxadustat ETTs. Moreover, roxadustat ETTs were shown to have a greater epithelial thickness than regular ETTs at early timepoints and valacyclovir ETTs at late timepoints. These findings contradict those of other groups who have investigated drug-loaded electrospun fiber coatings on ETTs and found reduced laryngotracheal mucosal thickness in comparison to regular ETTs; those studies employed a steroid formulation of mometasone [52, 53].

Following mechanical injury, macrophages respond to inflammatory signals in the microenvironment to restore homeostasis. Macrophages exhibit either pro-inflammatory (M1) or anti-inflammatory (M2) phenotypes depending on the stage of wound repair, and disproportionate polarization towards one phenotype has been associated with the pathogenesis of chronic conditions and disease [54]. In the intubated airway, M2 polarization has been linked to tracheostomy-associated granulation tissue and laryngotracheal stenosis [55–57]. To further complicate immune response to mucosal irritation from intubation, ETTs serve as a substrate for microorganisms to adhere to, and bacterial colonization can elicit macrophage dysfunction, dampening the inflammatory response necessary for biofilm clearance of subsequent infections. One instance of this is in the observation of *Staphylococcus aureus*, a gram-positive bacterium capable of causing biofilm infections on indwelling medical devices and has been shown to polarize macrophages toward an anti-inflammatory profibrotic phenotype [58, 59]. In the present study, immunostaining of CD86 and CD206, M1 and M2 surface markers, indicated a significant decrease of macrophages within the vocal fold epithelium over time with both roxadustat and valacyclovir ETT placement. Tissue with roxadustat ETT placement had a greater percentage of M2 macrophages in comparison to valacyclovir ETT treated tissue at early timepoints. Roxadustat is a hypoxia-inducible factor prolyl hydroxylase (HIF-PH) inhibitor that leads to accumulation of HIF-α. Two major isoforms of HIF-α are HIF-1α and HIF-2α, which have been shown to be expressed exclusively in M1 and M2 phenotypes, respectively [60]. There does not appear to be an inverse relationship between M1 and M2 macrophages in the injured airway treated with roxadustat ETT to verify which isoform of HIF-α is being activated in these groups; however. The finding of increased macrophages in the roxadustat ETTs correlates with the increased inflammation scores determined from pathologist assessment of H&E stained vocal fold tissue.

Cytokines also play an important role in immune response to injury and infection by coordinating macrophage activation and production. M1 macrophages are polarized by IFN-γ and TNF-α then produce higher levels of IL-1, IL-6, and IL-12. Alternatively, M2 macrophages are polarized by IL-4, IL-10, and TGF-β and generate increased expression of IL-10 and TGF-β [54, 61]. In injured laryngeal tissue, IL-6 had increased levels in comparison to healthy controls at 3 days corresponding to peak inflammatory response during wound healing [62]. The observed decrease in IL-6 levels over time likely reflects the resolution of inflammation and a shift toward an anti-inflammatory environment as tissue repair begins. The gradual upregulation of IL-4 over time is consistent with normal immune response following injury. Tissues with valacyclovir ETT placement demonstrated higher levels of many inflammatory cytokines than with roxadustat ETTs including IL-10, TNF-α, IFN-α, IL-1β, and IL-8. This trend suggests roxadustat, which is known to stabilize hypoxia, could be mitigating inflammation more effectively by promoting a faster resolution of the hypoxic environment after airway injury [14].

The upper respiratory tract has an important role in the mucociliary clearance pathway that is impaired upon ETT placement. This weakening of airway defense mechanisms has been identified as the pathogenesis for airway infections such as ventilated associated pneumonia (VAP) [63]. Antimicrobial-resistant pathogens commonly associated with VAP are *Enterococcus faecalis*, *Staphylococcus aureus, Klebsiella pneumoniae, Acinetobacter baumannii, Pseudomonas aeruginosa,* and *Enterobacter* species (ESKAPE) some of which have been regularly identified in ETT biofilms [64–66]. The correlation between specific microbial composition and airway etiologies extends beyond VAP. For instance, stenosis has been associated with distinct bacterial recognition of *Moraxella* and *Acinetobacter* and an inverse correlation of *Prevotella* and *Streptococcus* [67]. In the injured airway simulating traumatic intubation, roxadustat ETT placement showed negative fold changes in *Actinobacillus, Bacteroides, Bergeyella*, and *Dielma* microbes in comparison to regular ETT placement whereas valacyclovir ETTs showed an increase in *Phascolarctobacterium*. While SEM demonstrated copious bacterial adhesion and biofilm formation on the surface of all ETTs, µCT demonstrated that fiber coated ETTs had greater inclusions than uncoated (regular) ETTs. This finding likely results from the increased surface area in the fiber coated ETTs. Noteworthy microbes with increased log fold changes on the ETT surface were *Bacteroides, Filifactor, Fusobacterium, Peptococcus,* and *Prevotella_7* some of which are commensal bacteria that become opportunistic under specific conditions and have previously been detected in respiratory infection and diseases [68–72].

While our study provides valuable insights into the effects of localized therapeutic delivery on laryngeal injury, several limitations should be considered. Variability in the severity of scarring across samples, influenced by differences in airway injury and anatomical variations, may limit the generalizability of our findings. Although we aimed to optimize the number of animals used, the study was constrained by a small sample size. Additionally, while swine airways closely resemble human airways, species-specific responses, particularly in bacterial composition, may not fully translate to human conditions. Mechanical testing could have yielded more comprehensive data if different indentation depths and indenter tip diameters had been utilized, offering greater insight into tissue properties beyond the superficial layer. Freezing artifacts resulting from refreezing the larynges after biomechanical testing may have further obscured subtle histological changes. Moreover, our exclusion of other activators of M1 and M2 macrophages limits the scope of the immune response analysis. Future studies will explore these additional inflammatory stimuli and biologically active substances. Finally, to assess whether the early changes observed in this study contribute to long-term complications, such as stenosis, we aim to introduce long-term follow-ups into future investigations.

## Conclusion

Laryngeal injury and localized delivery of roxadustat and valacyclovir elicited alterations in the vocal fold mechanics, inflammatory response, and respiratory microbiota. Local stiffness outcomes were increased in the injured and intubated airway and inflammation appeared to influence bacterial composition and diversity in the upper respiratory microbiome, potentially impacting local tissue healing. These alterations were further influenced by the specific therapeutic agent administered. Our findings provide insight into the complex structural and molecular processes associated with wound healing and future investigations will aim to translate these insights into improving therapeutic options, optimizing laryngeal tissue recovery, and mitigating post-intubation complications effectively.

## Supplementary Information

Supplementary Material 1.

## Data Availability

No datasets were generated or analysed during the current study.
