# Supplementary material for: Localized delivery of therapeutics impact laryngeal mechanics, local inflammatory response, and respiratory microbiome following upper airway intubation injury in swine
Source: Respir Res. 2024 Sep 28;25:351. doi: 10.1186/s12931-024-02973-1 (PMC11439253; doi:10.1186/s12931-024-02973-1)
Supplement: Supplementary file 1 — Supplementary Material 1. [file 12931_2024_2973_MOESM1_ESM.docx]

**Supplementary Table 1.** Differential abundance of taxa in the airway with corresponding p-values and adjusted p-values.

| **Genus** | **3d- 0d** | | **7d- 0d** | | **14d- 0d** | | **Rox. - Reg.** | | **Val.- Reg.** | |
| --- | --- | --- | --- | --- | --- | --- | --- | --- | --- | --- |
|  | p value | adj  p value | p value | adj  p value | p value | adj  p value | p value | adj  p value | p value | adj  p value |
| *[Eubacterium] brachy group* | 0.377 | 1.000 | 0.132 | 1.000 | 0.846 | 1.000 | 0.098 | 1.000 | 0.549 | 1.000 |
| *[Eubacterium] nodatum group* | 0.075 | 1.000 | 0.122 | 1.000 | 0.000 | ***0.006** | 0.133 | 1.000 | 0.053 | 1.000 |
| *Acetitomaculum* | 0.481 | 1.000 | 0.843 | 1.000 | 0.727 | 1.000 | 0.009 | 0.546 | 0.239 | 1.000 |
| *Acinetobacter* | 0.045 | 1.000 | 0.812 | 1.000 | 0.625 | 1.000 | 0.271 | 1.000 | 0.390 | 1.000 |
| *Actinobacillus* | 0.445 | 1.000 | 0.428 | 1.000 | 0.698 | 1.000 | 0.000 | ***0.022** | 0.112 | 1.000 |
| *Actinomyces* | 0.002 | 0.124 | 0.133 | 1.000 | 0.007 | 0.299 | 0.015 | 0.865 | 0.012 | 0.715 |
| *Alloprevotella* | 0.524 | 1.000 | 0.047 | 1.000 | 0.037 | 1.000 | 0.022 | 1.000 | 0.454 | 1.000 |
| *Alysiella* | 0.470 | 1.000 | 0.685 | 1.000 | 0.393 | 1.000 | 0.741 | 1.000 | 0.009 | 0.557 |
| *Anaerovorax* | 0.852 | 1.000 | 0.027 | 1.000 | 0.000 | ***<0.0001** | 0.018 | 0.995 | 0.176 | 1.000 |
| *Atopobium* | 0.008 | 0.432 | 0.213 | 1.000 | 0.009 | 0.372 | 0.397 | 1.000 | 0.477 | 1.000 |
| *Bacteroides* | 0.001 | ***0.030** | 0.000 | ***0.020** | 0.000 | ***<0.0001** | 0.000 | ***<0.0001** | 0.154 | 1.000 |
| *Bergeriella* | 0.927 | 1.000 | 0.166 | 1.000 | 0.002 | 0.105 | 0.591 | 1.000 | 0.619 | 1.000 |
| *Bergeyella* | 0.020 | 0.954 | 0.874 | 1.000 | 0.564 | 1.000 | 0.000 | ***0.001** | 0.003 | 0.182 |
| *Blautia* | 0.758 | 1.000 | 0.182 | 1.000 | 0.099 | 1.000 | 0.930 | 1.000 | 0.197 | 1.000 |
| *Brachybacterium* | 0.405 | 1.000 | 0.280 | 1.000 | 0.310 | 1.000 | 0.113 | 1.000 | 0.393 | 1.000 |
| *Campylobacter* | 0.103 | 1.000 | 0.074 | 1.000 | 0.006 | 0.273 | 0.068 | 1.000 | 0.888 | 1.000 |
| *Catonella* | 0.000 | ***0.001** | 0.002 | 0.154 | 0.000 | ***<0.0001** | 0.262 | 1.000 | 0.121 | 1.000 |
| *Caviibacter* | 0.933 | 1.000 | 0.267 | 1.000 | 0.001 | ***0.031** | 0.562 | 1.000 | 0.457 | 1.000 |
| *Collinsella* | 0.800 | 1.000 | 0.203 | 1.000 | 0.106 | 1.000 | 0.141 | 1.000 | 0.064 | 1.000 |
| *Conchiformibius* | 0.489 | 1.000 | 0.099 | 1.000 | 0.001 | 0.056 | 0.541 | 1.000 | 0.441 | 1.000 |
| *Corynebacterium* | 0.188 | 1.000 | 0.083 | 1.000 | 0.262 | 1.000 | 0.074 | 1.000 | 0.083 | 1.000 |
| *Desulfovibrio* | 0.378 | 1.000 | 0.000 | ***0.020** | 0.000 | ***<0.0001** | 0.002 | 0.106 | 0.003 | 0.167 |
| *Dielma* | 0.004 | 0.204 | 0.984 | 1.000 | 0.997 | 1.000 | 0.000 | ***0.027** | 0.009 | 0.569 |
| *DNF00809* | 0.000 | ***0.014** | 0.099 | 1.000 | 0.019 | 0.725 | 0.016 | 0.909 | 0.004 | 0.251 |
| *Escherichia-Shigella* | 0.568 | 1.000 | 0.044 | 1.000 | 0.004 | 0.192 | 0.518 | 1.000 | 0.480 | 1.000 |
| *Faecalibacterium* | 0.447 | 1.000 | 0.174 | 1.000 | 0.230 | 1.000 | 0.746 | 1.000 | 0.162 | 1.000 |
| *Family XIII UCG-001* | 0.002 | 0.117 | 0.029 | 1.000 | 0.030 | 1.000 | 0.022 | 1.000 | 0.366 | 1.000 |
| *Filifactor* | 0.001 | ***0.045** | 0.002 | 0.126 | 0.000 | ***<0.0001** | 0.861 | 1.000 | 0.159 | 1.000 |
| *Fusobacterium* | 0.901 | 1.000 | 0.007 | 0.423 | 0.000 | ***0.003** | 0.082 | 1.000 | 0.221 | 1.000 |
| *Gemella* | 0.093 | 1.000 | 0.227 | 1.000 | 0.622 | 1.000 | 0.015 | 0.865 | 0.299 | 1.000 |
| *Globicatella* | 0.070 | 1.000 | 0.267 | 1.000 | 0.027 | 1.000 | 0.659 | 1.000 | 0.985 | 1.000 |
| *Helcococcus* | 0.057 | 1.000 | 0.020 | 1.000 | 0.005 | 0.219 | 0.134 | 1.000 | 0.141 | 1.000 |
| *HT002* | 0.979 | 1.000 | 0.795 | 1.000 | 0.276 | 1.000 | 0.805 | 1.000 | 0.367 | 1.000 |
| *Johnsonella* | 0.455 | 1.000 | 0.421 | 1.000 | 0.276 | 1.000 | 0.123 | 1.000 | 0.592 | 1.000 |
| *Kocuria* | 0.101 | 1.000 | 0.025 | 1.000 | 0.134 | 1.000 | 0.127 | 1.000 | 0.069 | 1.000 |
| *Lachnoclostridium* | 0.097 | 1.000 | 0.130 | 1.000 | 0.007 | 0.299 | 0.408 | 1.000 | 0.641 | 1.000 |
| *Lactobacillus* | 0.978 | 1.000 | 0.955 | 1.000 | 0.669 | 1.000 | 0.826 | 1.000 | 0.378 | 1.000 |
| *Leptotrichia* | 0.175 | 1.000 | 0.641 | 1.000 | 0.781 | 1.000 | 0.005 | 0.302 | 0.368 | 1.000 |
| *Macrococcus* | 0.961 | 1.000 | 0.997 | 1.000 | 0.566 | 1.000 | 0.513 | 1.000 | 0.468 | 1.000 |
| *Mageeibacillus* | 0.274 | 1.000 | 0.548 | 1.000 | 0.636 | 1.000 | 0.255 | 1.000 | 0.024 | 1.000 |
| *Micrococcus* | 0.228 | 1.000 | 0.244 | 1.000 | 0.307 | 1.000 | 0.121 | 1.000 | 0.332 | 1.000 |

**Supplementary Table 1 continued.**

| **Genus** | **3d- 0d** | | **7d- 0d** | | **14d- 0d** | | **Rox. - Reg.** | | **Val.- Reg.** | |
| --- | --- | --- | --- | --- | --- | --- | --- | --- | --- | --- |
|  | p value | adj  p value | p value | adj  p value | p value | adj  p value | p value | adj  p value | p value | adj  p value |
| *Moraxella* | 0.182 | 1.000 | 0.438 | 1.000 | 0.958 | 1.000 | 0.097 | 1.000 | 0.699 | 1.000 |
| *Murdochiella* | 0.000 | ***0.0001** | 0.001 | 0.080 | 0.000 | ***0.006** | 0.039 | 1.000 | 0.208 | 1.000 |
| *Neisseria* | 0.009 | 0.438 | 0.762 | 1.000 | 0.670 | 1.000 | 0.109 | 1.000 | 0.204 | 1.000 |
| *Oribacterium* | 0.712 | 1.000 | 0.348 | 1.000 | 0.037 | 1.000 | 0.498 | 1.000 | 0.877 | 1.000 |
| *Parvimonas* | 0.007 | 0.348 | 0.454 | 1.000 | 0.029 | 1.000 | 0.012 | 0.721 | 0.908 | 1.000 |
| *Peptoanaerobacter* | 0.665 | 1.000 | 0.719 | 1.000 | 0.514 | 1.000 | 0.184 | 1.000 | 0.173 | 1.000 |
| *Peptococcus* | 0.000 | ***0.004** | 0.001 | 0.070 | 0.000 | ***<0.0001** | 0.281 | 1.000 | 0.080 | 1.000 |
| *Peptoniphilus* | 0.003 | 0.169 | 0.008 | 0.495 | 0.098 | 1.000 | 0.995 | 1.000 | 0.400 | 1.000 |
| *Peptostreptococcus* | 0.006 | 0.296 | 0.044 | 1.000 | 0.009 | 0.371 | 0.580 | 1.000 | 0.229 | 1.000 |
| *Phascolarctobacterium* | 0.695 | 1.000 | 0.213 | 1.000 | 0.005 | 0.222 | 0.036 | 1.000 | 0.000 | ***<0.0001** |
| *Porphyromonas* | 0.020 | 0.954 | 0.054 | 1.000 | 0.001 | ***0.035** | 0.162 | 1.000 | 0.477 | 1.000 |
| *Prevotella* | 0.810 | 1.000 | 0.058 | 1.000 | 0.010 | 0.406 | 0.956 | 1.000 | 0.417 | 1.000 |
| *Prevotella_7* | 0.000 | ***0.014** | 0.036 | 1.000 | 0.000 | ***0.026** | 0.915 | 1.000 | 0.027 | 1.000 |
| *Prevotellaceae UCG-003* | 0.185 | 1.000 | 0.087 | 1.000 | 0.000 | ***0.026** | 0.348 | 1.000 | 0.006 | 0.384 |
| *Proteocatella* | 0.000 | ***0.007** | 0.838 | 1.000 | 0.819 | 1.000 | 0.928 | 1.000 | 0.493 | 1.000 |
| *Rothia* | 0.072 | 1.000 | 0.170 | 1.000 | 0.246 | 1.000 | 0.143 | 1.000 | 0.176 | 1.000 |
| *S5-A14a* | 0.014 | 0.674 | 0.018 | 1.000 | 0.000 | ***0.004** | 0.369 | 1.000 | 0.578 | 1.000 |
| *Staphylococcus* | 0.488 | 1.000 | 0.939 | 1.000 | 0.312 | 1.000 | 0.062 | 1.000 | 0.089 | 1.000 |
| *Stomatobaculum* | 0.000 | ***0.002** | 0.051 | 1.000 | 0.002 | 0.110 | 0.096 | 1.000 | 0.140 | 1.000 |
| *Streptobacillus* | 0.028 | 1.000 | 0.379 | 1.000 | 0.360 | 1.000 | 0.001 | 0.067 | 0.002 | 0.103 |
| *Streptococcus* | 0.749 | 1.000 | 0.197 | 1.000 | 0.582 | 1.000 | 0.033 | 1.000 | 0.955 | 1.000 |
| *Subdoligranulum* | 0.803 | 1.000 | 0.268 | 1.000 | 0.100 | 1.000 | 0.535 | 1.000 | 0.127 | 1.000 |
| *Treponema* | 0.000 | ***0.019** | 0.002 | 0.143 | 0.000 | ***<0.0001** | 0.775 | 1.000 | 0.879 | 1.000 |
| *Trueperella* | 0.119 | 1.000 | 0.097 | 1.000 | 0.003 | 0.146 | 0.152 | 1.000 | 0.330 | 1.000 |
| *Tyzzerella* | 0.000 | ***0.006** | 0.084 | 1.000 | 0.002 | 0.099 | 0.086 | 1.000 | 0.929 | 1.000 |
| *Veillonella* | 0.730 | 1.000 | 0.590 | 1.000 | 0.709 | 1.000 | 0.052 | 1.000 | 0.960 | 1.000 |
| *Weissella* | 0.605 | 1.000 | 0.228 | 1.000 | 0.833 | 1.000 | 0.803 | 1.000 | 0.797 | 1.000 |

**Table S1.** Airway swabs taken at 3, 7, and 14 days post-injury were compared to pre-injury swabs (0 days) using Analysis of Compositions of Microbiomes with Bias Correction (ANCOM-BC2). Additionally, differential abundance was assessed between airway swabs from Roxadustat- and Valacyclovir-eluting ETT placement compared to Regular ETTs.

**Supplementary Table 2.** Differential abundance of taxa on the surface of ETTs with corresponding p-values and adjusted p-values.

| **Genus** | **3d- 0d** | | **7d- 0d** | | **14d- 0d** | | **Rox. - Reg.** | | **Val.- Reg.** | |
| --- | --- | --- | --- | --- | --- | --- | --- | --- | --- | --- |
|  | p value | adj  p value | p value | adj  p value | p value | adj  p value | p value | adj  p value | p value | adj  p value |
| *[Eubacterium] nodatum group* | 0.036 | 0.861 | 0.031 | 0.493 | 0.000 | ***0.010** | 0.167 | 1.000 | 0.190 | 1.000 |
| *Acinetobacter* | 0.002 | 0.063 | 0.001 | ***0.032** | 0.000 | ***0.006** | 0.321 | 1.000 | 0.370 | 1.000 |
| *Actinobacillus* | 0.042 | 0.970 | 0.000 | ***0.010** | 0.000 | ***0.006** | 0.005 | 0.254 | 0.505 | 1.000 |
| *Actinomyces* | 0.000 | ***0.001** | 0.001 | ***0.042** | 0.000 | ***0.005** | 0.077 | 1.000 | 0.326 | 1.000 |
| *Alloprevotella* | 0.712 | 1.000 | 0.581 | 1.000 | 0.216 | 1.000 | 0.061 | 1.000 | 0.985 | 1.000 |
| *Anaerovorax* | 0.577 | 1.000 | 0.005 | 0.108 | 0.000 | ***<0.0001** | 0.042 | 1.000 | 0.506 | 1.000 |
| *Atopobium* | 0.000 | ***0.009** | 0.021 | 0.384 | 0.009 | 0.155 | 0.813 | 1.000 | 0.367 | 1.000 |
| *Bacteroides* | 0.000 | ***0.006** | 0.000 | ***<0.0001** | 0.000 | ***0.001** | 0.000 | ***0.001** | 0.051 | 1.000 |
| *Bergeriella* | 0.668 | 1.000 | 0.014 | 0.265 | 0.017 | 0.248 | 0.427 | 1.000 | 0.354 | 1.000 |
| *Bergeyella* | 0.047 | 1.000 | 0.008 | 0.153 | 0.009 | 0.155 | 0.002 | 0.093 | 0.005 | 0.225 |
| *Campylobacter* | 0.090 | 1.000 | 0.120 | 1.000 | 0.212 | 1.000 | 0.079 | 1.000 | 0.149 | 1.000 |
| *Catonella* | 0.000 | ***0.014** | 0.001 | ***0.041** | 0.000 | ***0.007** | 0.927 | 1.000 | 0.822 | 1.000 |
| *Conchiformibius* | 0.935 | 1.000 | 0.042 | 0.552 | 0.049 | 0.494 | 0.011 | 0.508 | 0.110 | 1.000 |
| *Corynebacterium* | 0.013 | 0.335 | 0.006 | 0.134 | 0.004 | 0.085 | 0.275 | 1.000 | 0.367 | 1.000 |
| *Desulfovibrio* | 0.149 | 1.000 | 0.002 | 0.063 | 0.000 | ***<0.0001** | 0.012 | 0.540 | 0.032 | 1.000 |
| *Dielma* | 0.010 | 0.257 | 0.002 | ***0.048** | 0.002 | ***0.045** | 0.004 | 0.193 | 0.007 | 0.353 |
| *DNF00809* | 0.000 | ***0.0003** | 0.000 | ***0.008** | 0.001 | ***0.028** | 0.038 | 1.000 | 0.311 | 1.000 |
| *Escherichia-Shigella* | 0.420 | 1.000 | 0.800 | 1.000 | 0.359 | 1.000 | 0.659 | 1.000 | 0.582 | 1.000 |
| *Falsiporphyromonas* | 0.009 | 0.246 | 0.000 | ***0.001** | 0.000 | ***<0.0001** | 0.369 | 1.000 | 0.341 | 1.000 |
| *Filifactor* | 0.000 | ***0.001** | 0.000 | ***<0.0001** | 0.000 | ***<0.0001** | 0.378 | 1.000 | 0.907 | 1.000 |
| *Fusobacterium* | 0.347 | 1.000 | 0.000 | ***0.004** | 0.000 | ***0.0002** | 0.031 | 1.000 | 0.514 | 1.000 |
| *Globicatella* | 0.013 | 0.329 | 0.003 | 0.077 | 0.003 | 0.061 | 0.646 | 1.000 | 0.402 | 1.000 |
| *Helcococcus* | 0.006 | 0.182 | 0.000 | ***0.007** | 0.026 | 0.357 | 0.505 | 1.000 | 0.062 | 1.000 |
| *Johnsonella* | 0.125 | 1.000 | 0.603 | 1.000 | 0.132 | 1.000 | 0.058 | 1.000 | 0.647 | 1.000 |
| *Kocuria* | 0.000 | ***0.0002** | 0.000 | ***<0.0001** | 0.000 | ***<0.0001** | 0.138 | 1.000 | 0.581 | 1.000 |
| *Lachnoclostridium* | 0.059 | 1.000 | 0.069 | 0.690 | 0.127 | 1.000 | 0.918 | 1.000 | 0.964 | 1.000 |
| *Mageeibacillus* | 0.912 | 1.000 | 0.779 | 1.000 | 0.932 | 1.000 | 0.493 | 1.000 | 0.019 | 0.923 |
| *Micrococcus* | 0.006 | 0.182 | 0.002 | 0.067 | 0.002 | ***0.049** | 0.141 | 1.000 | 0.700 | 1.000 |
| *Moraxella* | 0.001 | ***0.021** | 0.000 | ***0.006** | 0.000 | ***0.001** | 0.063 | 1.000 | 0.241 | 1.000 |
| *Murdochiella* | 0.000 | ***<0.0001** | 0.000 | ***0.002** | 0.000 | ***<0.0001** | 0.597 | 1.000 | 0.968 | 1.000 |
| *Neisseria* | 0.002 | 0.069 | 0.001 | ***0.033** | 0.000 | ***0.005** | 0.182 | 1.000 | 0.140 | 1.000 |
| *Oribacterium* | 0.960 | 1.000 | 0.118 | 1.000 | 0.000 | ***0.005** | 0.523 | 1.000 | 0.773 | 1.000 |
| *Parvimonas* | 0.000 | ***0.011** | 0.006 | 0.134 | 0.033 | 0.392 | 0.009 | 0.417 | 0.071 | 1.000 |
| *Peptoanaerobacter* | 0.330 | 1.000 | 0.876 | 1.000 | 0.802 | 1.000 | 0.097 | 1.000 | 0.051 | 1.000 |
| *Peptococcus* | 0.000 | ***0.002** | 0.000 | ***<0.0001** | 0.000 | ***<0.0001** | 0.801 | 1.000 | 0.919 | 1.000 |
| *Peptostreptococcus* | 0.000 | ***0.007** | 0.004 | 0.090 | 0.003 | 0.060 | 0.346 | 1.000 | 0.595 | 1.000 |
| *Porphyromonas* | 0.005 | 0.167 | 0.004 | 0.106 | 0.000 | ***0.012** | 0.151 | 1.000 | 0.702 | 1.000 |
| *Prevotella* | 0.761 | 1.000 | 0.062 | 0.685 | 0.003 | 0.071 | 0.816 | 1.000 | 0.316 | 1.000 |
| *Prevotella_7* | 0.000 | ***0.0002** | 0.000 | ***0.003** | 0.000 | ***0.006** | 0.711 | 1.000 | 0.604 | 1.000 |
| *Prevotellaceae UCG-003* | 0.116 | 1.000 | 0.054 | 0.650 | 0.000 | ***0.010** | 0.971 | 1.000 | 0.156 | 1.000 |
| *Proteocatella* | 0.003 | 0.101 | 0.025 | 0.423 | 0.037 | 0.404 | 0.632 | 1.000 | 0.107 | 1.000 |
| *Rothia* | 0.000 | ***<0.0001** | 0.000 | ***<0.0001** | 0.000 | ***<0.0001** | 0.344 | 1.000 | 0.427 | 1.000 |

**Supplementary Table 2 continued.**

| **Genus** | **3d- 0d** | | **7d- 0d** | | **14d- 0d** | | **Rox. - Reg.** | | **Val.- Reg.** | |
| --- | --- | --- | --- | --- | --- | --- | --- | --- | --- | --- |
|  | p value | adj  p value | p value | adj  p value | p value | adj  p value | p value | adj  p value | p value | adj  p value |
| *S5-A14a* | 0.002 | 0.071 | 0.000 | ***0.001** | 0.000 | ***0.002** | 0.594 | 1.000 | 0.986 | 1.000 |
| *Staphylococcus* | 0.112 | 1.000 | 0.270 | 1.000 | 0.069 | 0.625 | 0.543 | 1.000 | 0.215 | 1.000 |
| *Stomatobaculum* | 0.000 | ***0.001** | 0.000 | ***0.007** | 0.000 | ***0.001** | 0.121 | 1.000 | 0.376 | 1.000 |
| *Streptobacillus* | 0.082 | 1.000 | 0.038 | 0.535 | 0.029 | 0.375 | 0.006 | 0.295 | 0.003 | 0.135 |
| *Streptococcus* | 0.820 | 1.000 | 0.000 | ***0.005** | 0.000 | ***<0.0001** | 0.402 | 1.000 | 0.228 | 1.000 |
| *Treponema* | 0.059 | 1.000 | 0.000 | ***0.001** | 0.000 | ***0.0002** | 0.205 | 1.000 | 0.169 | 1.000 |
| *Trueperella* | 0.070 | 1.000 | 0.034 | 0.510 | 0.004 | 0.084 | 0.029 | 1.000 | 0.489 | 1.000 |
| *Tyzzerella* | 0.000 | ***0.002** | 0.000 | ***0.004** | 0.013 | 0.213 | 0.208 | 1.000 | 0.385 | 1.000 |
| *Veillonella* | 0.605 | 1.000 | 0.776 | 1.000 | 0.135 | 1.000 | 0.794 | 1.000 | 0.752 | 1.000 |

**Table S2.** Swabs of the surface of the endotracheal tubes (ETTs) were collected at 3, 7, and 14 days and compared to baseline swabs taken from the airway at 0 days (prior to injury and ETT placement) using ANCOM-BC2. Additionally, differential abundance was analyzed between swabs from Roxadustat- and Valacyclovir- eluting ETTs compared to Regular ETTs.
